# Neuroprotective effect of neuroserpin in non-tPA-induced intracerebral hemorrhage mouse models

**DOI:** 10.1186/s12883-017-0976-1

**Published:** 2017-11-07

**Authors:** Wei Li, Tetsuya Asakawa, Sha Han, Baoguo Xiao, Hiroki Namba, Chuanzhen Lu, Qiang Dong, Liang Wang

**Affiliations:** 1 Department of Neurology, Huashan Hospital, Fudan University, 12 Wulumuqi Zhong Road, Shanghai, 200040 People’s Republic of China; 2 Institute of Neurology, Huashan Hospital, Fudan University, Shanghai, China; 3 Huashan Worldwide Medical Center, Huashan Hospital, Fudan University, Shanghai, China; 4 Department of Neurosurgery, Hamamatsu University, School of Medicine, Handayama, 1-20-1, Higashi-ku, Hamamatsu-city, Shizuoka, 431-3192 Japan

**Keywords:** Neuroserpin, Intracerebral hemorrhage (ICH), Blood-brain barrier (BBB), Neuroprotective effect, Vascular endothelial cells, BBB permeability

## Abstract

**Background:**

The neuroprotective effects of neuroserpin (NSP) have been well documented in both patients and animal models with cerebral ischemia; however, have never been investigated in hemorrhagic stroke. The aim of this study is to verify the neuroprotection of NSP in the non-tPA-induced intracerebral hemorrhage (ICH) mouse model.

**Methods:**

C57BL/6J male mice (*n* = 198) were involved in this study. ICH models were established with infusion of autologous blood into the brain parenchyma. We then detected NSP expression in ICH brains by morphological methods and western blotting analysis. We measured the brain water content and detected blood-brain barrier (BBB) permeability to verify the neuroprotective effects of NSP.

**Results:**

We found that NSP protein expression was upregulated in ICH models, with a peak at 48 h after ICH induction. NSP local administration reduced the brain edema and the BBB permeability in ICH models. The neurological deficits were also ameliorated. Thus, the neuroprotection of NSP in ICH state was confirmed. Additionally, we also found that the distribution pattern of occludin-expressing cells was obviously changed by the ICH procedure but partly recovered after NSP administration. This finding indicated that protecting and/or repairing the injured vascular endothelial cells may be a potential mechanism involved in NSP neuroprotection, which needs further verification.

**Conclusions:**

Our results supported the fact that NSP may be considered as a potential therapy for ICH for the neuroprotective effects including amelioration of the edema.

**Electronic supplementary material:**

The online version of this article (10.1186/s12883-017-0976-1) contains supplementary material, which is available to authorized users.

## Background

Neuroserpin (NSP), as an inhibitor of the tissue plasminogen activator (tPA), has been reported to exert neuroprotective effects in animal models [1–3] and in patients with cerebral ischemia (CI) [4]. The detailed mechanism involved in the neuroprotection of NSP is unclear. Our previous study showed the potential efficacy of NSP in the pathogenesis and treatment of CI [2]. Later studies indicated that the mechanisms may be related to preventing N-methyl-D-aspartic acid-induced neurotoxicity [5], reducing tPA-mediated inflammation and disruption of the blood-brain barrier (BBB) [6], thus destroying the balance between NSP and tPA expression [1]. Our recent study showed that NSP has a neuroprotective effect on ischemic astrocytes [7] neurons, microglia [8], and in patients [9]. The neuroprotection on ischemic brain and/or cells is confirmed regardless of the detailed mechanism being uncertain. We hypothesized that such neuroprotection also exists on a hemorrhagic brain.

Intracerebral hemorrhage (ICH) is a common type of stroke. Although the pathophysiology of ICH is quite different than that of CI, they almost share the same pathogenesis [10]. ICH can be induced by inappropriate intravenous tPA therapy in CI individuals, leading to failure of the BBB, thus collapsing cerebral capillaries that can no longer hold blood constituents and contributing to the formation of an edema [11]. Another recent report indicated that tPA increases the neurological deficits in tPA-induced ICH [12]. It is understandable that NSP is capable of reducing the incidence of hemorrhagic transformation induced by tPA thrombolysis in experimental CI models, contributing to an increase in the therapeutic window for tPA thrombolysis [13, 14]. Nevertheless, with regard to the non-tPA-related hemorrhage, insights toward the role of endogenous tPA are limited. Whether NSP has a neuroprotective effect in non-tPA-induced ICH brains through a tPA-induced or non-tPA-induced mechanism is interesting and may be useful to develop a new treatment strategy of ICH. In this context, the primary task of the present study is to verify whether NSP shows its neuroprotective effects on a non-tPA-induced hemorrhagic brain, such as attenuating the BBB permeability ameliorating the edema, then improving the neurological deficits. We therefore observed whether NSP can reverse these neurotoxic effects induced by an experimental ICH process. Moreover, we observed the distribution pattern of occludin-expressing cells to investigate effects of NSP on vascular endothelial cells. We believe these experiments are helpful to achieve a deeper understanding of whether NSP has neuroprotective effects on non-tPA-induced ICH. To the best of our knowledge, this is the first study that focused on the effects of NSP in a non-tPA-induced hemorrhagic stroke.

## Methods

### Establishment and verification of the ICH model

**Fig. 1:**
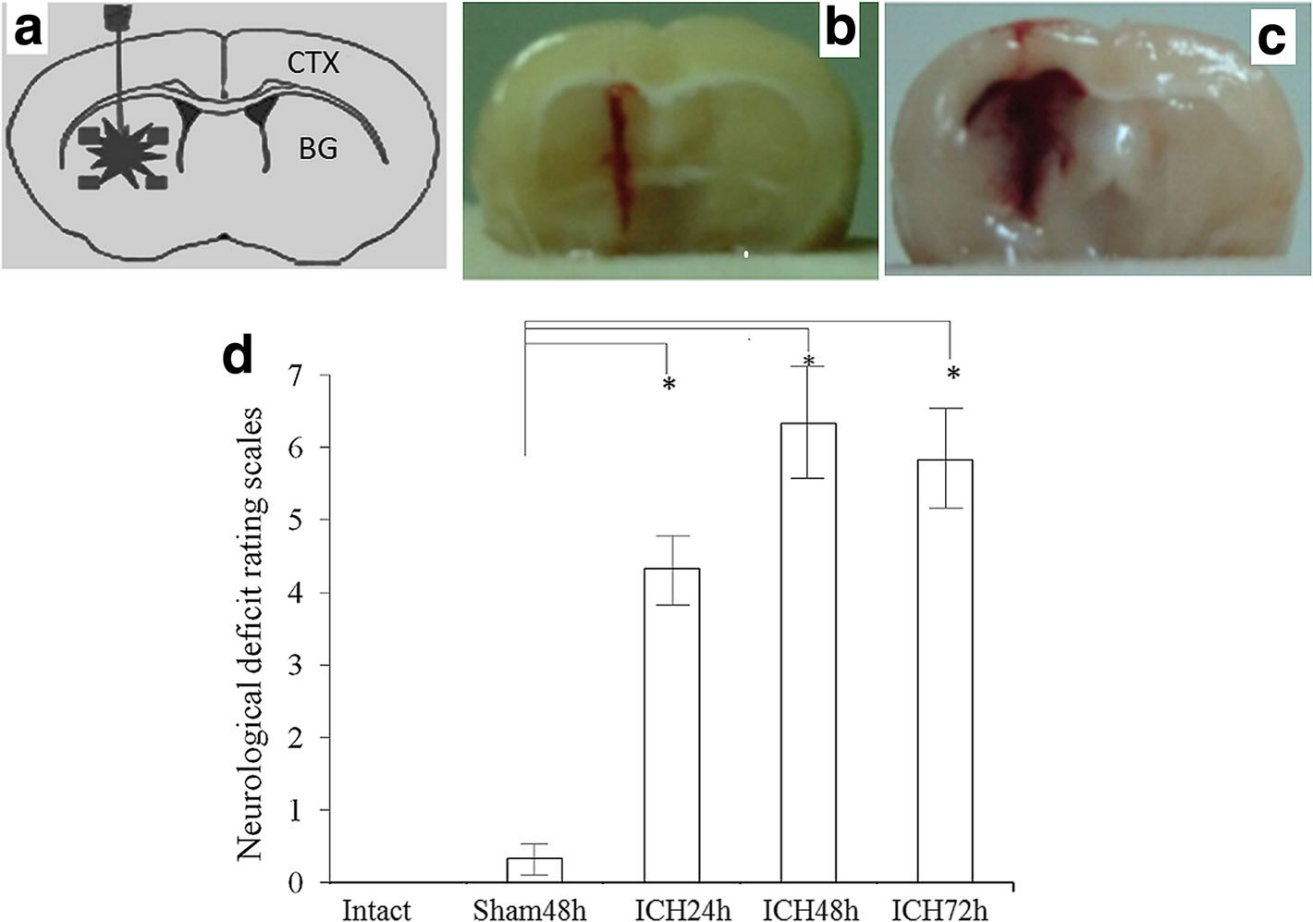
Establishment of the intracerebral hemorrhage (ICH) mouse model. **a**. The location of the blood injection. **b**. Coronal section of a sham mouse. **c**. Coronal section of an ICH model. **d**. Behavioral assessments of the ICH model. We found that the ICH procedure significantly increased the neurological deficit rating scores (ICH groups vs. sham group), while the symptoms reached a peak 48 h after the surgery (ICH48h vs. ICH24h). *, *P* < 0.05. CTX: cortex; BG: basal ganglia; R: right; L: left

A total of 198 male C57BL/6J mice (8–12 weeks old, body weight 25–35 g; Shanghai Institute of the Chinese Academy of Science, China) were involved in this study. All animals were treated according to the National Institute of Health Guidelines for the Care and Use of Laboratory Animals. All experimental procedures were approved by the Animal Care and Use Committee of the Fudan University (authorization No: 0556262). After anesthetization with 10% chloral hydrate [400 mg/kg, Sinopharm Chemical Reagent Co. (SCRC), Ltd., China], the ICH model was established after double-injection of autologous blood (15 μL) into the brain parenchyma (basal ganglia) of the mice, as described previously [15] (Fig. 1a). ICH mice were divided into three groups according to the duration of ICH procedure: ICH24h (*n* = 36), ICH48h (*n* = 60), and ICH72h (*n* = 36). We also used sham models (*n* = 54, 36 for experiments and 18 for the pre-experiments) inserting the injection needle into the basal ganglia without injection of the autologous blood. We used sham models with injection needle into the basal ganglia. In our pre-experiments, we compared the neurological deficit rating scales, Numbers of NSP + expression cells, and NSP expression in the rats of sham24h, shame48h and sham72h (*n* = 6 respectively), and found there were no significant difference of neurological deficit (Additional file 1: Figure S1), Numbers of NSP + expression cells (Additional file 2: Figure S2A and S2B) among these sham models in different time points. We therefore used sham 48 h as our standard sham models in the present study. The blank control mice (*n* = 12) were sacrificed without any surgery, deeply anesthetized using chloral hydrate (400 mg/kg).

To verify the success of these ICH models, a rating scale (RS), based on a battery measuring the neurological function in mice, described previously [16] was adopted to assess the neurological deficits of the animals. Briefly, two tasks, i.e., the postural reflex test (PRT) and the forelimb placing test (FPT) were included in RS. The scores of PRT are 0–2 according to the different levels of deficits of the upper body posture while the animal was held by the tail. The scores of FPT included three subitems measuring the sensorimotor integration when forelimbs were placed responding to visual (dorsal placing 0–2; lateral placing 0–2), tactile (dorsal placing 0–2; lateral placing 0–2), and proprioceptive (0–2) stimuli. The maximal scores are 12 by adding the scores of PRT and FPT, indicating the most severe deficits [17]. The behavioral assessments were performed by an independent staff blinded to the other experiments to avoid the observation bias [18, 19].After finishing the behavioral test, the animals (blank control, *n* = 12; sham48h, *n* = 12; ICH24h, *n* = 12; ICH48h, *n* = 12; ICH72h, *n* = 12) were deeply anesthetized by an overdose of chloral hydrate (400 mg/kg), the brains were removed and placed in formalin solution until slicing. The brains were dehydrated in a 10% sucrose solution for 1 day and then 30% sucrose solution for 2–3 days, till the brain sank to the bottom of the bottle [20]. Then, 10-μm coronal sections were cut on a freezing microtome and processed for confirmation of the hematoma; staining and western blotting analyses were conducted to detect NSP expression.

### Detection of NSP expression in ICH brains by IHC + Hoechst double staining and western blotting analysis

The abovementioned mice (*n* = 6 in each group) were used in this analysis. Brains were stained following standard IHC procedures described previously [1]. In brief, sections from 2.5 to 3.5 mm from the bregma were selected. All sections were first immersed in methanol 0.3% H_2_O_2_ in the dark, treated with 0.3% Triton X-100 phosphate-buffered saline (PBS), and then preincubated with 10% goat serum (CWBIO, China) for 20 min at 23 °C. Then, they were incubated with a primary rabbit anti-NSP antibody (ab33077, 1:100; Abcam, USA) overnight at 4 °C; the control group was incubated with 10% PBS instead. A secondary goat anti-rabbit IgG-fluorescein isothiocyanate (FITC) antibody (CW0114, 1:100; CWBIO) was added and incubated for 60 min at 37 °C. Then for double staining, a standard Hoechst staining was employed to observe changes in nuclear DNA of these slices [21]. Hoechst 33,342 (Sigma B2261, 1:5000) was added and incubated for 10 min. Sections were covered with 50% glycerin and dried, and pictures were taken using a light microscope (Olympus, Japan) and a digital camera (Olympus). NSP-expressing cells were identified by the green staining in the cells with blue staining in the nuclear DNA. We counted the NSP-expressing cells in each slice and got the average.

A standard western blotting procedure was employed to analyze NSP expression [1]. The mice mentioned above (*n* = 6 in each group) were involved in this analysis. Briefly, brain homogenates were prepared using RIPA/ phenylmethanesulfonylfluoride or phenylmethylsulfonyl fluoride (PMSF) (100 μL/1 μL with 10-mg brain tissue) buffer and pulsed with ultrasound. They were frozen in ice for 30 min and then centrifuged for 20 min at 12,000 rpm at 4 °C. The supernatants were separated and boiled with a 5× loading buffer for 5 min at 100 °C and then frozen in ice. NSP protein in the supernatants was separated by 10% sodium dodecyl sulfate polyacrylamide gel electrophoresis and transferred to a polyvinylidene fluoride membrane. Blots were incubated with a primary rabbit anti-NSP antibody (ab33077, 1:250, Abcam), and a mouse anti-β tubulin antibody (KC5701, 1:10,000, Kangchen, China) overnight at 4 °C. According to user manual of the kits, the sensitivity is 1–2 μg/mL, and the antibodies are specific for neuroserpin without any significant cross reactivity or interference with serpin A6, serpin I2 (both at 100 ng/mL), serpin A12 (at 2 ng/ mL), urinary trypsin inhibitor (at 100 ng/ mL) and S100B (at 4 ng/ mL) in mice. After incubating with a secondary goat anti-rabbit antibody (CW0103, 1:5000, CWBIO) and goat anti-mouse antibody (CW0102, 1:10,000, CWBIO) coupled with horseradish peroxidase (HRP), antigen–antibody reaction bands were visualized using a chemiluminescence detection kit (Thermo Scientific, USA), recorded, and quantified using an Image-Pro Plus 5.0 software (Media Cybernetics, USA).

### Verifying the neuroprotective effects of NSP with measurements of the behavioral performance, brain water content and the BBB permeability

ICH24h, ICH48h, ICH72h, and sham groups (*n* = 12 in each group) were involved in this experiment. The mice were anesthetized with 10% chloral hydrate (400 mg/kg), and recombinant human NSP (PeproTech, USA) (3 μL of 25 μg/mL) was stereotactically injected into the shallow cortex of the hematoma (AP = 1 mm; LP = 2 mm; depth = 3 mm from bregma), making the ICH + NSP group. The control groups using ICH24h, ICH48h, ICH72h (*n* = 12 in each group), received 3 μL of saline (0.9%, also given to the sham group). When animals recovered from the anesthesia, RS [16] was performed to assess the locomotor abilities of all animals in each group. Once the behavioral assessments were completed, the animals were deeply anesthetized with 10% chloral hydrate (400 mg/kg) for the next experiments.

Half of the mice mentioned above (*n* = 6 in each group) were involved in this experiment. The brains were removed and brain tissues (100 mg) around the hematoma were excised, and the wet weight was accurately measured. Then, all of the samples were dried in an oven (for 24 h at 100 °C, Zuofei, China). The dry weight was also measured accurately. Brain water content was calculated as (wet weight − dry weight)/wet weight × 100%.

Evens Blue (EB, SCRC LTD., China) was diluted to a 2% solution with 0.9% sterilized saline. Then the solution was injected into the caudal veins of the remaining mice (*n* = 6 in each group, 4 mL/ kg of body weight). The EB stain was allowed to circulate for 1 h following transcardially perfusion of 100 mL PBS. The brains were removed and the right hemispheres were selected as samples. All the samples were homogenized in methanamide (1 mL/100 mg of brain tissue), and then incubated overnight at 4 °C. The homogenates were then centrifuged (1000 rpm for 5 min at 4 °C, Thermo, USA), and the supernatants were collected. EB leakage was measured by a spectrophotometer (Multiskan MK3, Saimo, China) (λ = 632 nm) and quantified using a standard curve.

### IHC and Hoechst double staining for the occludin-expressing cells

Only the ICH48h group was involved in the following studies (ICH48h mice showed the maximal abnormality, see Results section). We made the ICH48h + NSP group (*n* = 12) along with the control group (ICH48h,*n* = 12) as we described in the above section. Both the control and sham groups received 3 μL of 0.9% saline. When the animals recovered from the anesthesia, the same RS [16] was used to confirm the motor deficits. Once the ICH symptoms were confirmed, the animals were again deeply anesthetized with 10% chloral hydrate (400 mg/kg) for the next experiment.

We used the same standard IHC staining procedures used for NSP to detect the distribution of occludin-expressing cells. We used an anti-occludin antibody (Invitrogen 331,500, 1:100, Invitrogen, USA) as the primary antibody, whereas goat anti-rabbit IgG-FITC (CW0114, 1:100, CWBIO) as the secondary antibody for IHC staining. Hoechst staining was performed for these slices undergoing double staining using Hoechst 33,342 (Sigma B2261, 1:5000). Photos were taken using a light microscope (Olympus, Japan) and a digital camera (Olympus, Japan).

### Statistical analysis

All statistical analyses were performed using SPSS software (V 13.0.0., SPSS, USA). To verify the difference among the control group, sham group, and ICH groups, data were analyzed using two-way ANOVA, followed by Bonferroni post-hoc correction; to compare the difference between groups with NSP treatment and groups without NSP administration, the Student’s t-test was used. Data are presented as mean ± standard error. *P* < 0.05 was accepted as statistically significant.

## Results

### Verification of the ICH model

Figure 1 shows the results of the establishing the ICH model. Figure 1b is a photo in a sham mouse. From a coronal section in an ICH model (Fig. 1c), the hematoma can be clearly observed. The behavioral data also showed that ICH significantly reduced motor performance (Fig. 1d). By morphological and behavioral evidence, we confirmed successfully establishing the ICH model.

### NSP expression in ICH brains

**Fig. 2:**
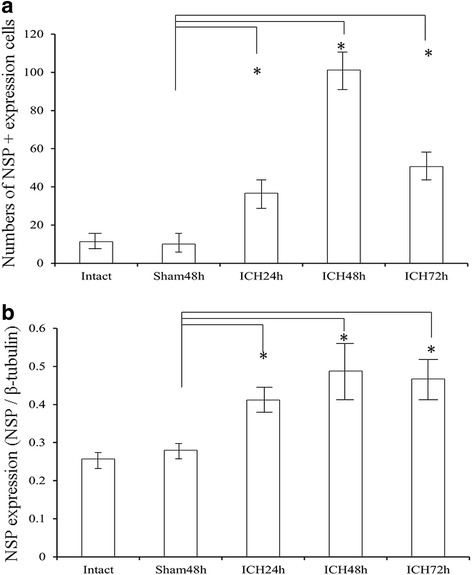
NSP expression in ICH brains. **a**. Quantitative results of IHC + Hoechst double staining for NSP-expression cells. Cell counting of the ICH model. We found that the ICH procedure significantly increased the numbers of NSP-expressing cells (ICH groups vs. sham48h group, while NSP-expressing cells reached a peak in the ICH48h group. *, *P* < 0.05. **b**. NSP protein expression in the tissues around the hematoma at 24 h/48 h/72 h after ICH. The quantitative results of western blotting for the NSP protein expression. We found that the bands of ICH24h, ICH48h, and ICH72h groups were stronger than those of the intact and sham48h groups during western blotting analysis (ICH groups vs. sham48h group), while no significant difference was found among the ICH groups (**b**). *, *P* < 0.05

Figure 2a demonstrates the quantitative results of IHC + Hoechst double staining. We found NSP + expression cells in ICH groups were significantly higher than the sham48h group. Interestingly, we found that NSP expression reached a peak in the ICH48h group (Fig. 2a), consistent with the behavioral performance. The worst rating scores were found in the ICH48h (Fig. 1d). The figure of IHC + Hoechst staining is available in Additional file 2: Figure S2C.

Western blotting analysis was conducted for quantitatively analyzing NSP protein expression. NSP protein expression was upregulated in the ICH groups in comparison with the sham48h group (Fig. 2b). However, we did not find any difference among the ICH groups.

Our findings demonstrated that NSP protein expression was upregulated in ICH.

### Verifying the neuroprotective effects of NSP by local administration

**Fig. 3:**
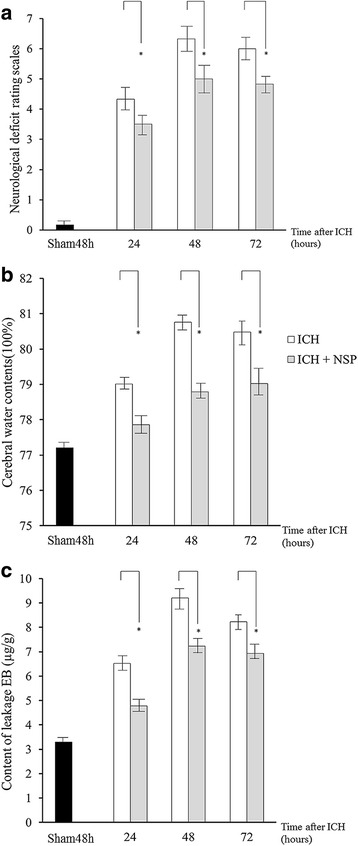
NSP local administration showed neuroprotective effect for ICH. Sham48h group: black column; ICH group: open column; ICH + NSP group: gray column. *, *P* < 0.05. **a**. NSP local administration significantly reduced the neurological deficit rating scores (ICH vs. ICH + NSP), but never reaching a normal level. **b**. NSP local administration significantly reduced the water content in the ICH brains. (ICH vs. ICH + NSP). **c**. NSP local administration significantly reduced EB leakage in the ICH brains (ICH vs. ICH + NSP)

Figure 3 demonstrates the efficacy of NSP local administration. The behavioral performance (Fig. 3a), the cerebral water content (Fig. 3b), and the EB leakage (Fig. 3c) were significantly ameliorated by NSP administration. Our data showed that NSP reduced brain edema and the BBB permeability in the ICH state. Accordingly, the motor performance was also significantly improved. By these data, we verified the neuroprotective effects of NSP in an ICH rat brain.

### Changes of the distribution pattern of occludin-expressing cells related to NSP local administration

**Fig. 4:**
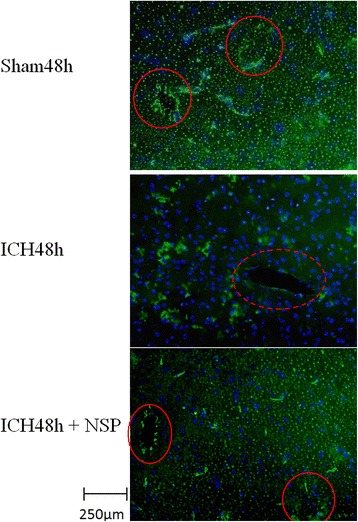
Morphological assays for occludin-expressing cells by IHC + Hoechst. In the sham48h group, occludin-expressing cells (bright green spots) distributed regularly, surrounding the capillaries (the distribution pattern is shown with a solid red ring), whereas in the ICH group, we could not find this pattern; the distribution of occludin-expressing cells became irregular. No occludin-expressing cell was found surrounding the capillaries (dotted red ring). After NSP administration (ICH48h + NSP), the occludin-expressing cells redistributed surrounding the capillaries, and the distribution pattern was observed again (solid red ring)

Figure 4 shows distribution of the occludin-expressing cell related to NSP local administration with IHC + Hoechst double staining. In the sham group, occludin-expressing cells were distributed regularly and formed a pattern surrounding the capillaries. On the other hand, in the ICH groups, this distribution could not be observed because no occludin-expressing cell was found surrounding the capillaries. Interestingly, in the ICH brains with NSP administration, the distribution pattern was re-established, with occludin-expressing cells around the capillaries. These figures indicated the changes of integrality of blood vessel endothelium affected by ICH procedure and NSP administration.

## Discussion

The most important finding in the present study is that we confirmed the neuroprotective effect of NSP in a non-tPA-induced ICH mouse brain. Edema and the enhanced BBB permeability caused by experimental ICH procedure were significantly ameliorated by NSP local administration. The related neurological deficits were also significantly improved. To the best of our knowledge, this is the first report that demonstrates the neuroprotection of NSP on non-tPA-induced hemorrhagic stroke. In addition, we found that the distribution pattern of occludin-expressing cells was recovered after NSP administration This finding gives us a hint that NSP may protect and/or repair blood vessel endothelium injury caused by ICH, which needs deeper investigation.

NSP is an inhibitor of tPA whose neuroprotective effects have been widely regarded in ischemic stroke. The present study verified our hypothesis of its neuroprotective effect in non-tPA-induced ICH. We selected an ICH model injecting blood directly into the brain, which showed delayed and progressive BBB disruption after 12–48 h [22, 23]. Our results showed edema (Fig. 3b) and disruption of the BBB (Fig. 3c) in ICH brains. Importantly, the severity of these changes showed the same tendency as the behavioral assessments (Fig. 1d and Fig. 3a); in particular, 48 h after ICH the mice showed the most severe motor deficits, the strongest edema, and the highest BBB permeability during the present experimental system. These phenomena do not exhibited in ischemic animal models. We believe that this is caused by the progression of hematoma, which is accordance with the neurologic deterioration occurs within 24 to 48 h after onset of ICH in human [24]. These pathological changes caused by ICH can be significantly improved by NSP local administration, regardless of the fact that the efficacy is limited and cannot reach a normal level (ICH + NSP group vs. sham48h group, Fig. 3). In this context, our results of NSP expression affected by the experimental ICH procedure can be easily understood. Because NSP showed a neuroprotective effect in ICH brains, the expression and distribution of NSP may be considered as a compensatory response after the ICH procedure (Fig. 2), which is analogous to what happens in ischemic brains [25], namely both hemorrhagic models in the present study and the ischemic models in the previous study [25] exhibit the peak NSP expression at 48 h after onset. Therefore, the upregulation of NSP expression after ICH may be an indirect evidence of the neuroprotective effect of NSP. There is no data regarding the half-life of NSP after being administrated to the animal. However, a previous study observed the changes 6 h after NSP administration in rat model of ischemic stroke [14], which indicated the long half-life of NSP, and our results were reliable.

Once the neuroprotection of NSP was verified, we supposed that NSP may play a role in protection of the BBB integrity, because the BBB permeability can be reduced by NSP administration. We therefore investigated the BBB integrity by observing the occludin-expressing cells. Occludin is a TJ integral membrane protein expressed at the cell - cell junctions of epithelial and endothelial cells [12]. The BBB comprises specialized cerebral microvascular endothelial cells connected by TJs to form an impermeable monolayer devoid of transcellular pores [13]. Occludin is indispensable for maintaining the barrier integrity in various endothelial cell models. It is reported that the quality of the endothelial barrier is directly correlated with the level of occludin expression [14]. Downregulation of occludin is directly linked to elevated endothelial permeability [15]. Another report suggested that not only the structure but also the localization of occludin is crucial to preserve the function of BBB to restrict permeability to solutes in the systemic circulation. Any alterations in the localization and structure of occludin may damage the BBB [16]. We found that experimental ICH procedure changes the distribution pattern of occludin-expressing cells, which can be re-established by the following NSP local administration. The occludin-expressing cells normally surround the capillaries, and the BBB also works normally. Then the distribution of occludin-expressing cells was damaged by the ICH procedure, indicated the integrality of blood vessel endothelium were damaged, while the simultaneous experiments showed the enhancement of BBB permeability, edema, and motor deficits. However, after treatment by NSP administration, occludin-expressing cells were observed surrounding the capillaries (Fig. 4), indicating that the BBB was partly repaired and/or protected. Meanwhile, we also achieved reduced BBB permeability and palliative edema and improved behavioral performance. NSP may be efficient to protect vascular endothelial cells, allowing occludin-expressing cells to redistribute surrounding the vascular wall. In the present study, we have only described this phenomenon. We will further investigate the changes in more details, including the changes of vascular endothelial cells and other TJs in our future study.

**Fig. 5:**
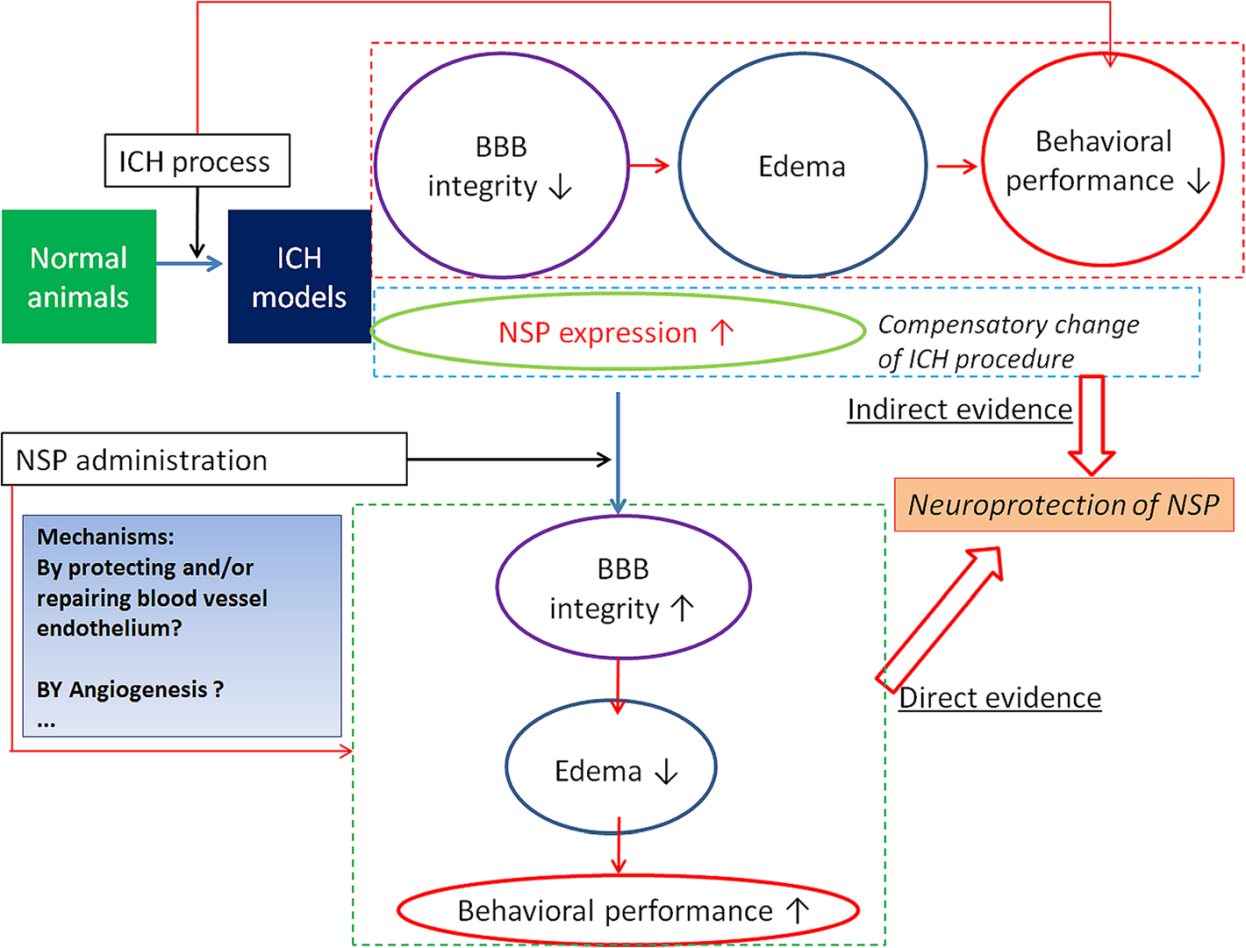
The potential mechanism of neuroprotective effect of NSP in non-tPA-induced ICH

Taken together, we speculated the potential mechanisms of neuroprotective effect of NSP on non-tPA-induced ICH in Fig. 5 based on the findings of the present study. NSP may play a role in repairing the blood vessel endothelium (or by angiogenesis), which results in improving the BBB integrity, and then relieving edema, and finally improve the behavioral performance. Meanwhile, the high NSP expression induced by ICH processes provides indirect evidence for the neuroprotection of NSP. Furthermore, the present study does not use a tPA-induced ICH model; we still do not know the role of endogenous tPA in an acute ICH state. Because endogenous tPA is regulated by its endogenous inhibitor, NSP [13], compensatory upregulation of NSP suggests that the endogenous tPA plays a role in the neurotoxic effects of ICH, regardless of a previous study reporting that the role of endogenous tPA appears to be limited to the early phase of edema formation [26]. Consequentially, the present study could not determine whether the neuroprotective effect of NSP is related to tPA or not. Our future experiment will use a tPA knockout mouse ICH model to investigate if the neuroprotective effect of NSP is related to the endogenous tPA, which is important to achieve a better understanding of the roles of endogenous tPA in an acute ICH state.

Although this study provides evidence for the neuroprotection of NSP on non-tPA –induced ICH in rodent models, the roles of NSP in management of hemorrhagic stroke in humans, namely the clinical values of NSP are still unknown. Our recent study in patients with ischemic stroke proved the neuroprotective effects of NSP, and we concluded that Serum NSP level can be considered as potential predictive factors for outcome of acute ischemic stroke [9]. We speculated that NSP also exhibits neuroprotective effects in patients with hemorrhagic stroke. Therefore, whether administration of NSP can improve the neurological deficits in ICH patients; whether serum can be a predict factor for outcomes of ICH? Such questions should be verified in our future investigations.

## Conclusions

As a conclusion, we verified the neuroprotective effect of NSP in non-tPA-induced ICH brains. NSP can be considered as a potential adjuvant therapy, which reduces brain edema through attenuating the permeability of the BBB possibly by protecting and/or repairing the injured blood vessel endothelium in the ICH state.

## Additional files

Additional file 1: Figure S1. Neurological deficit rating scales in the sham models in different time points. We did not find any significant difference among the Sham24h, Sham48h and Sham72h groups. (TIFF 102 kb)

Additional file 2: Figure S2. Morphological assays results of NSP + expressing cells. A. NSP-expressing cells in the sham groups of different time points. B. We did not find any significant difference of the NSP-expressing cells among the Sham24h, Sham48h and Sham72h groups. C. We found that NSP-expressing cells were upregulated in the tissues surrounding the hematoma (green areas). NSP-expressing cells were prominent near the superficial areas of the hematoma. Arrow shows a typical NSP-expressing cell; white line shows the range of the hematoma; * shows the area of the hematoma. (TIFF 1671 kb)

## Abbreviations

BBB: blood brain barrier
CI: cerebral ischemia
EB: Evens Blue
FPT: forelimb placing test
HRP: horseradish peroxidase
ICH: intracerebral hemorrhage
NSP: neuroserpin
PMSF: phenylmethylsulfonyl fluoride
PRT: postural reflex test
TJ: tight junction
tPA: tissue plasminogen activator
