# Supplementary figures and images for: Neuroprotective effect of neuroserpin in non-tPA-induced intracerebral hemorrhage mouse models
Source: BMC Neurol. 2017 Nov 7;17:196. doi: 10.1186/s12883-017-0976-1 (PMC5688810; doi:10.1186/s12883-017-0976-1)

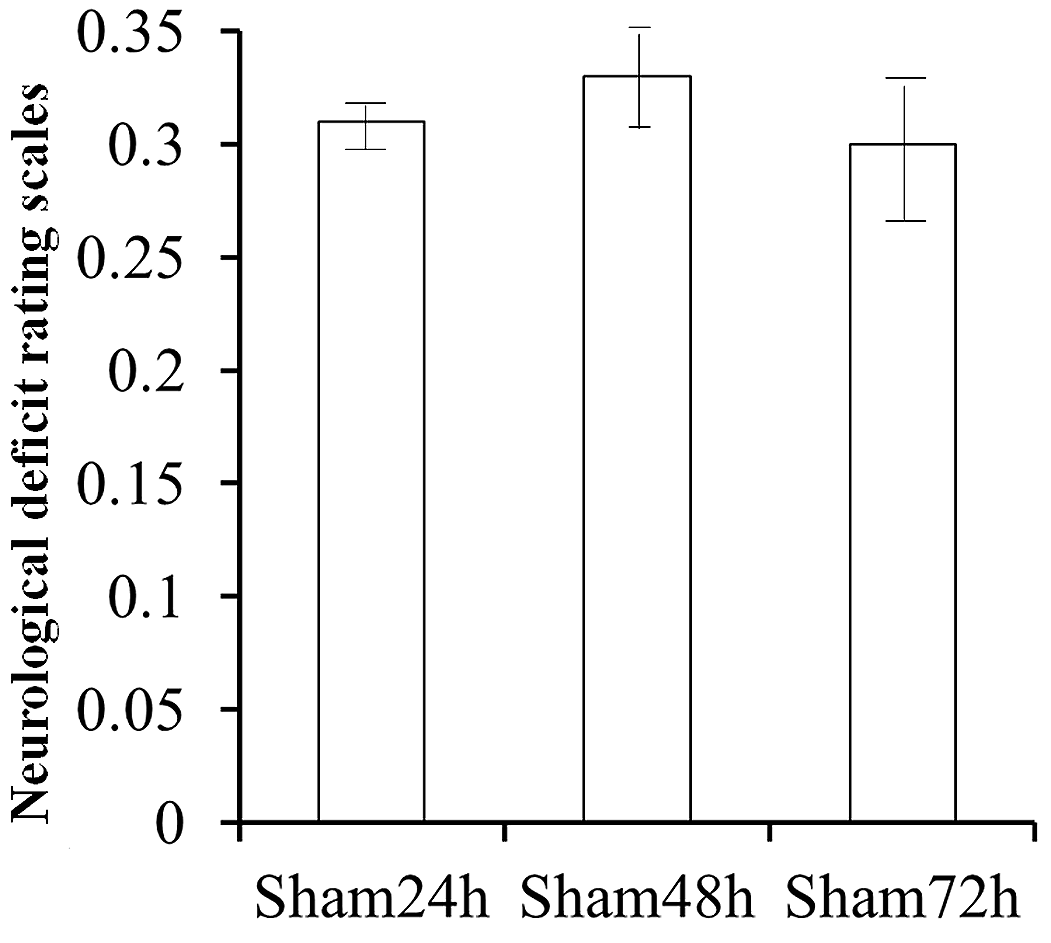

Supplement: Supplementary file 1 — Neurological deficit rating scales in the sham models in different time points. We did not find any significant difference among the Sham24h, Sham48h and Sham72h groups. (TIFF 102 kb) [file 12883_2017_976_MOESM1_ESM.tif]

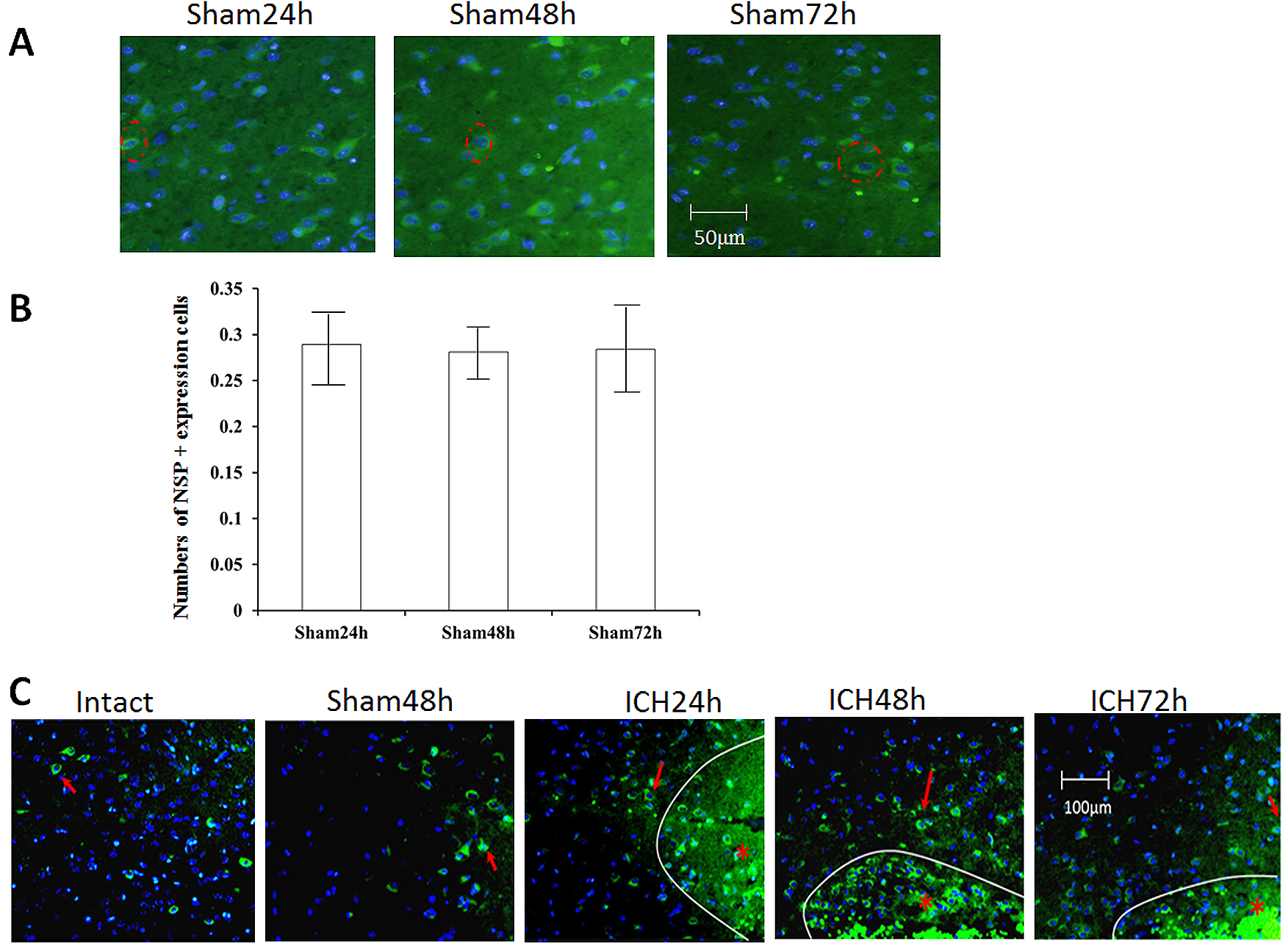

Supplement: Supplementary file 2 — Morphological assays results of NSP + expressing cells. A. NSP-expressing cells in the sham groups of different time points. B. We did not find any significant difference of the NSP-expressing cells among the Sham24h, Sham48h and Sham72h groups. C. We found that NSP-expressing cells were upregulated in the tissues surrounding the hematoma (green areas). NSP-expressing cells were prominent near the superficial areas of the hematoma. Arrow shows a typical NSP-expressing cell; white line shows the range of the hematoma; * shows the area of the hematoma. (TIFF 1671 kb) [file 12883_2017_976_MOESM2_ESM.tif]
